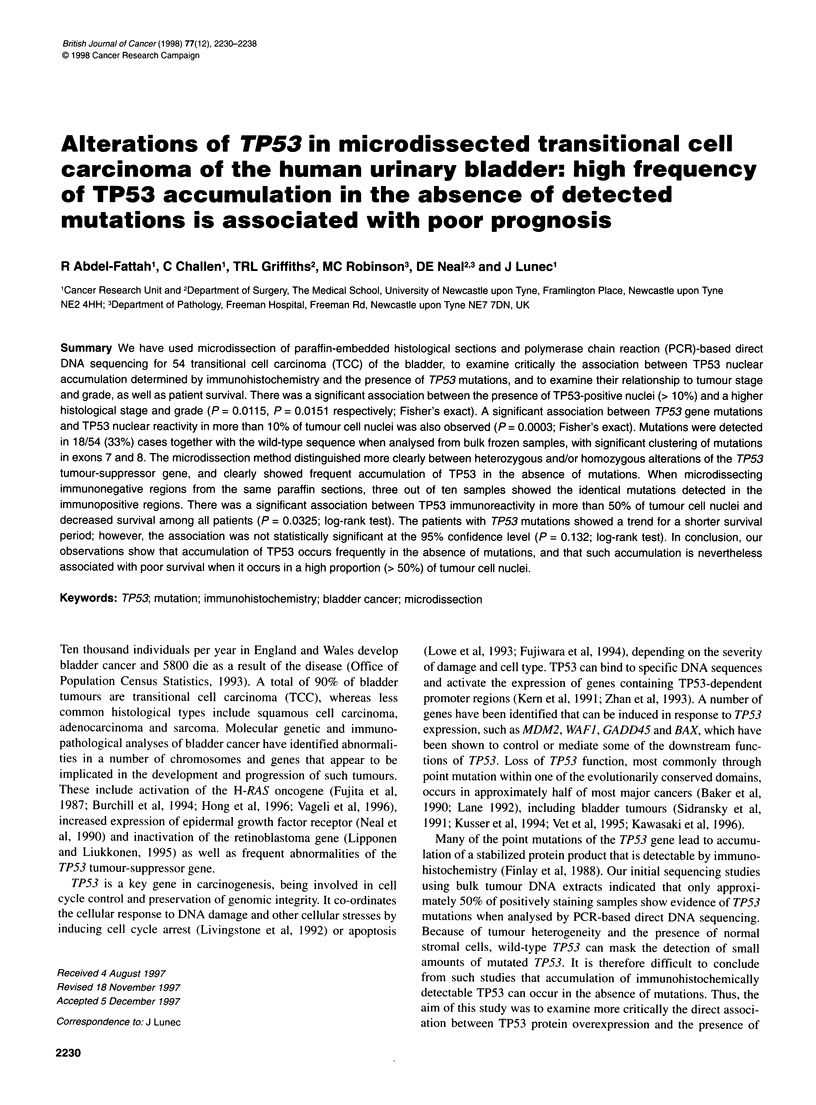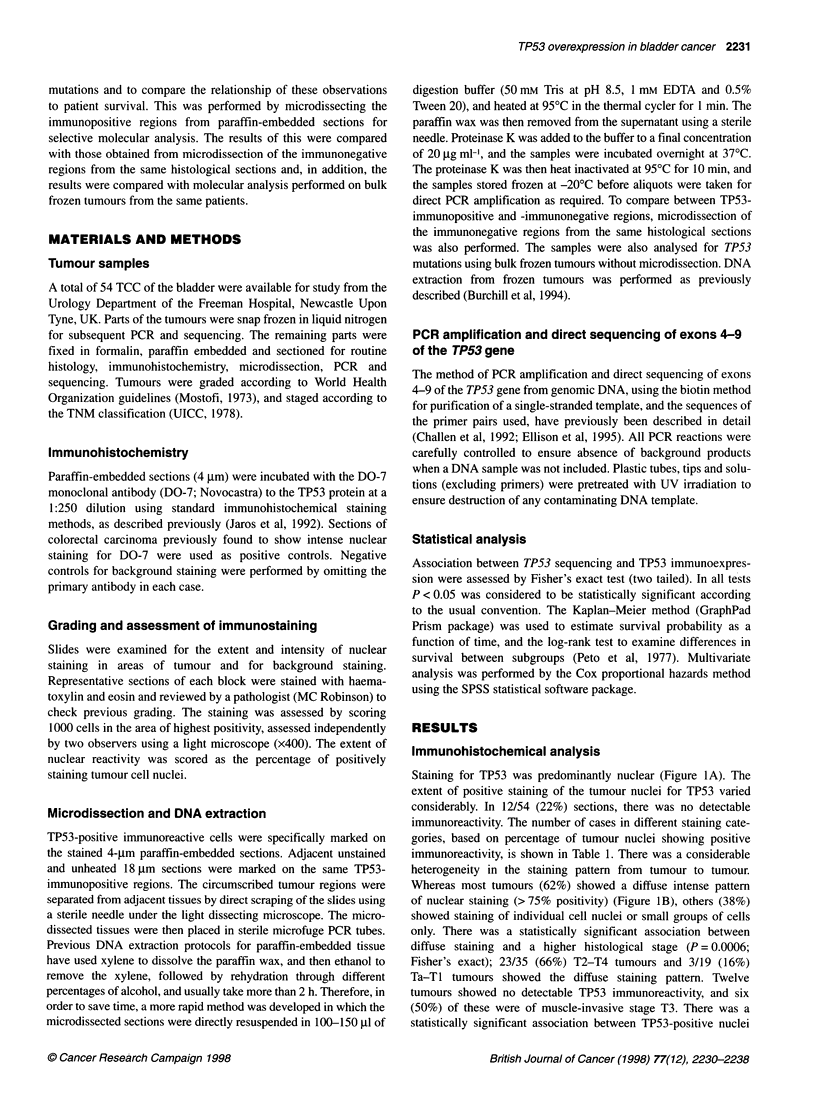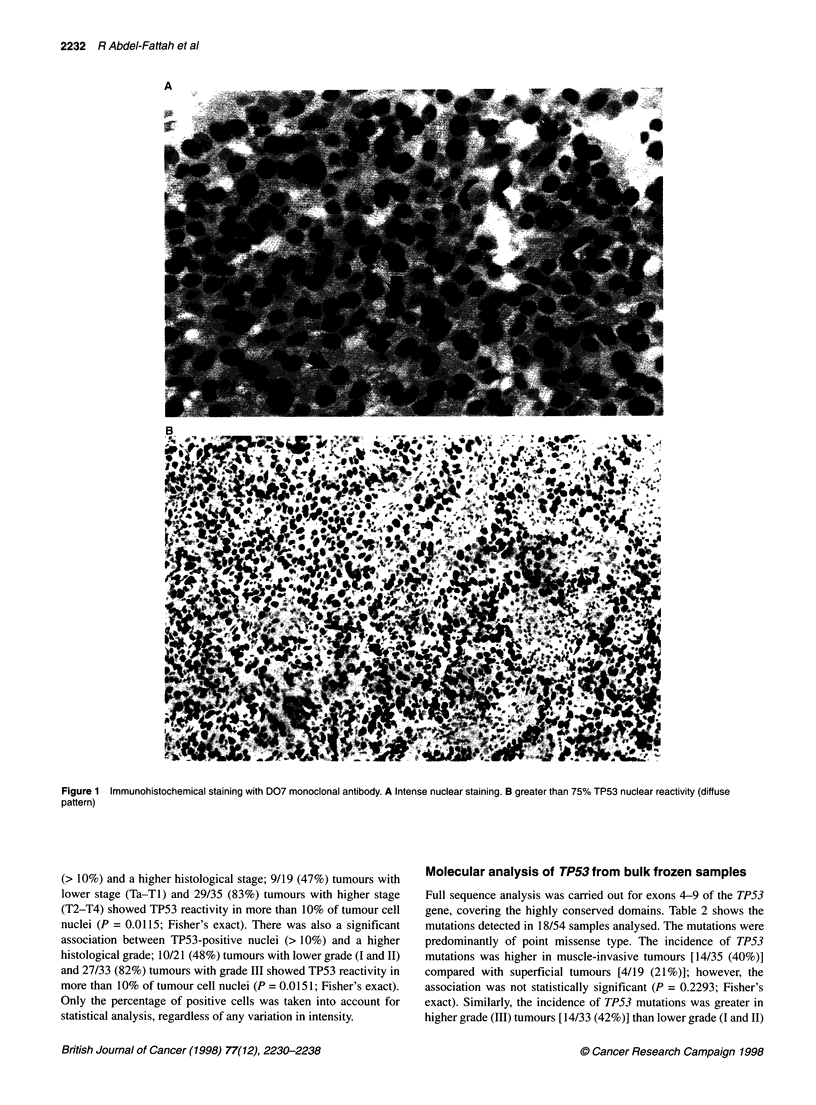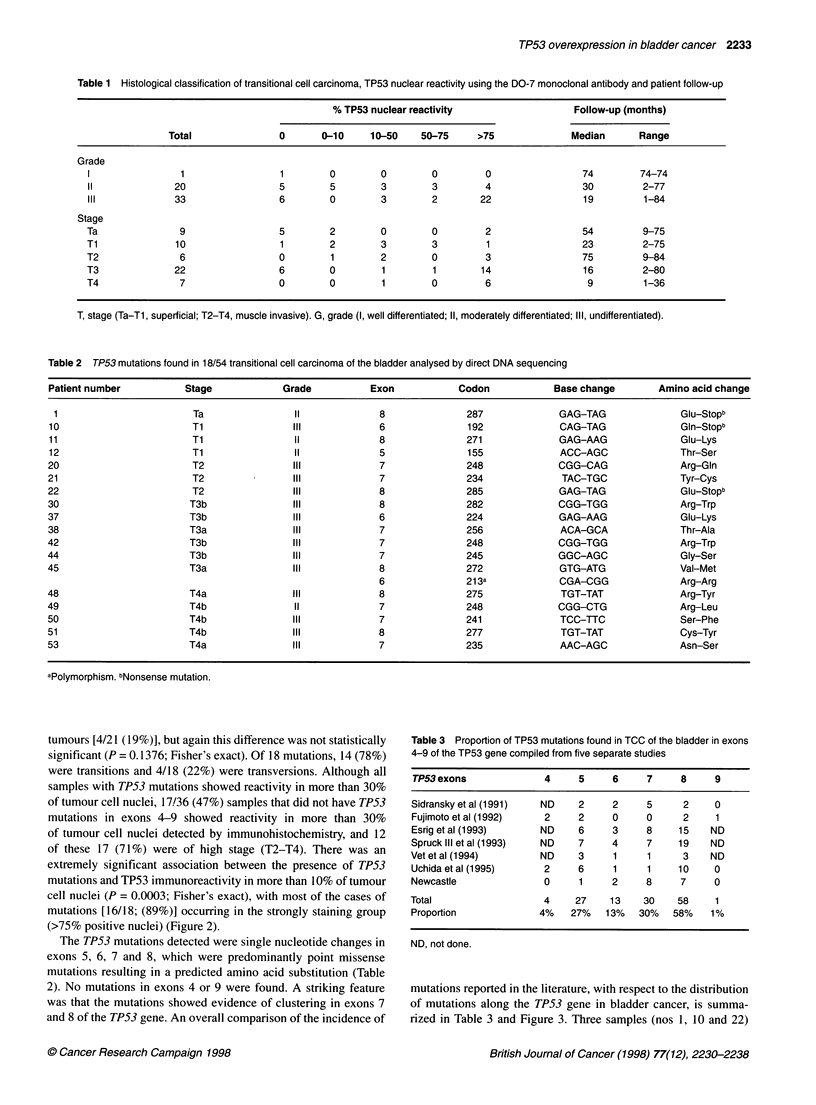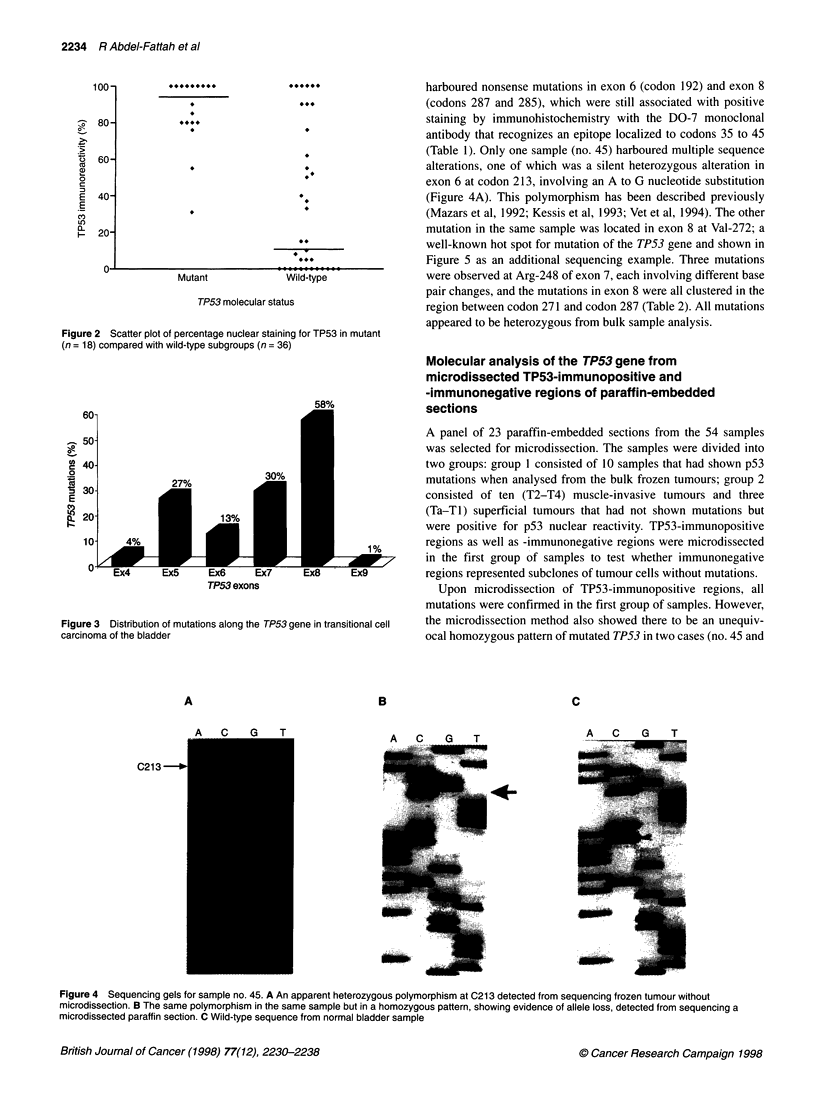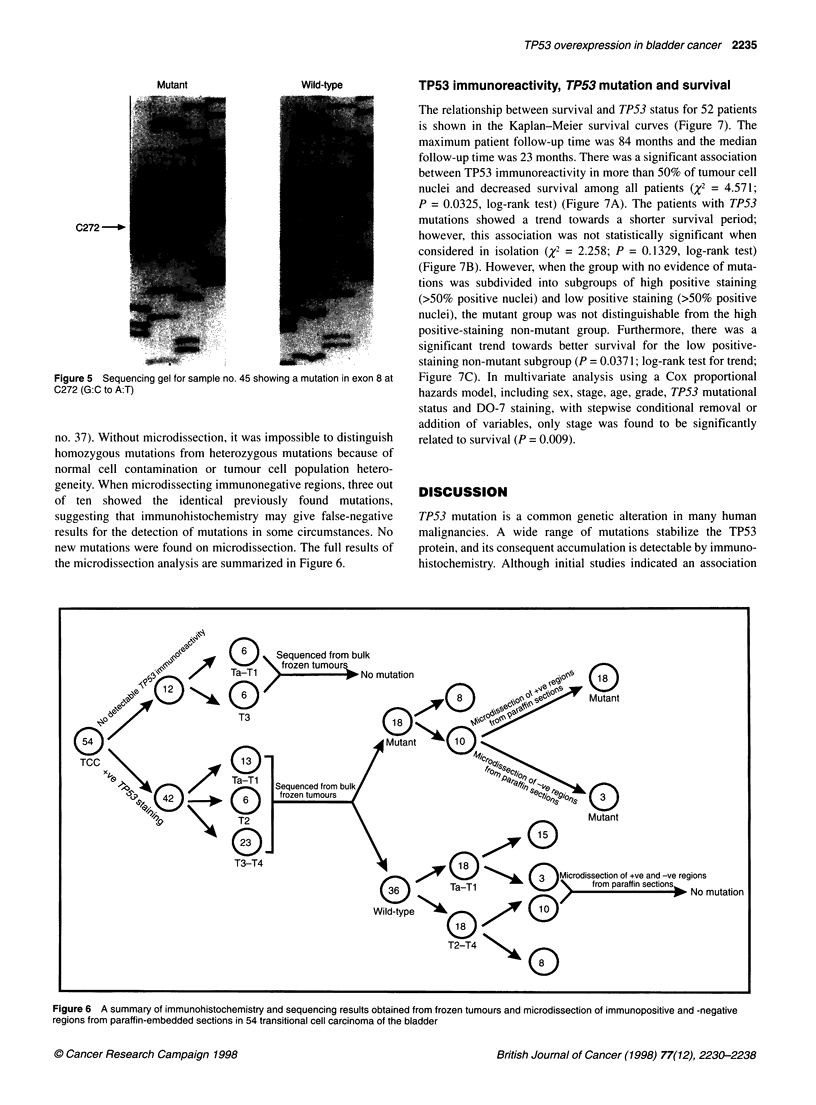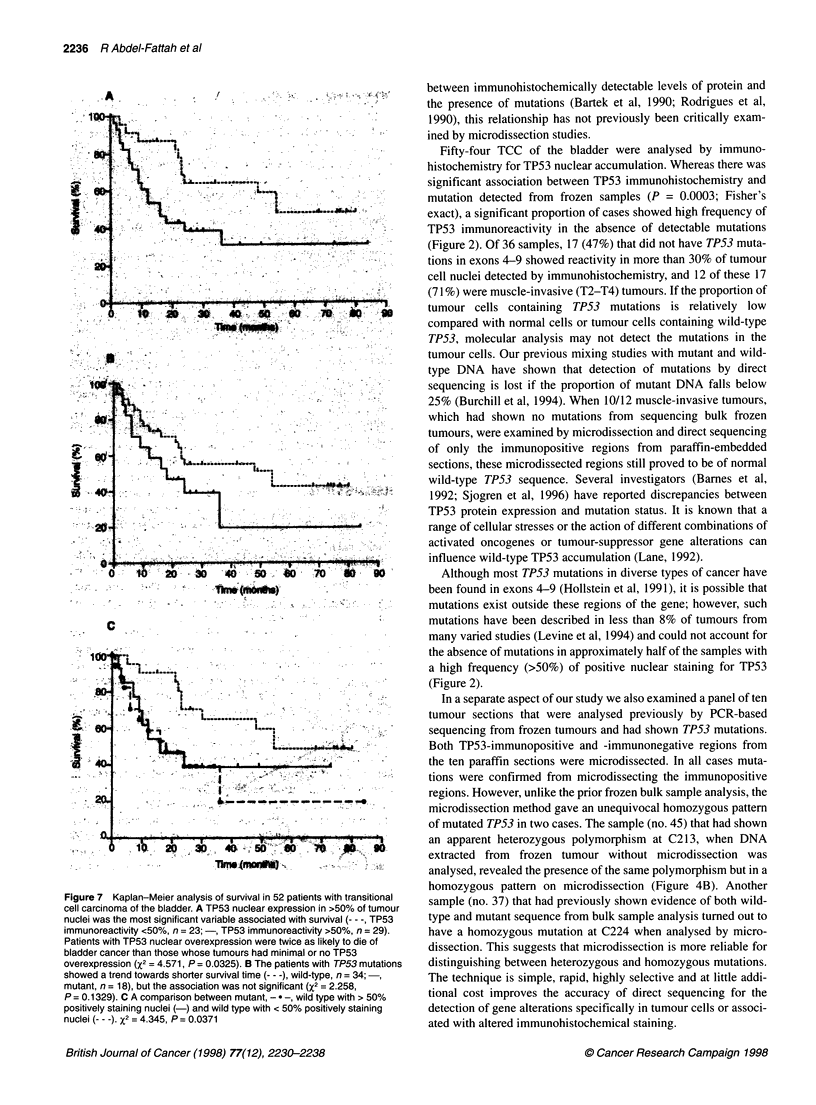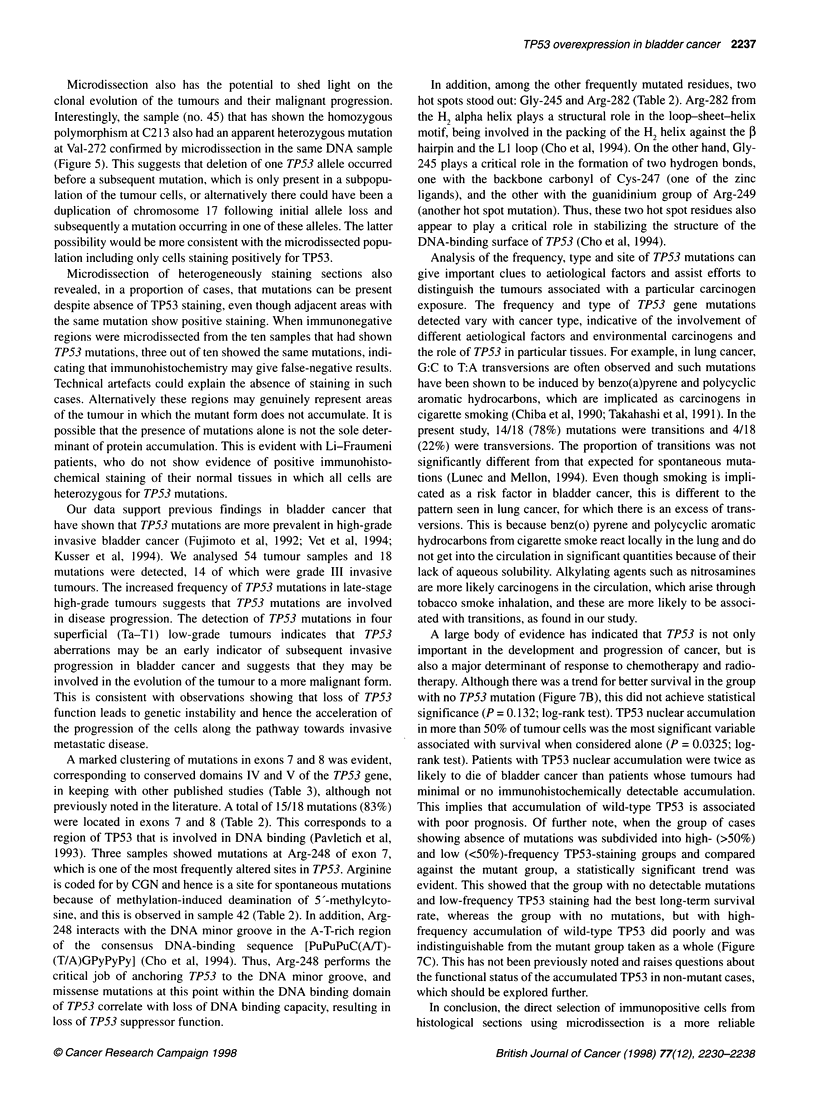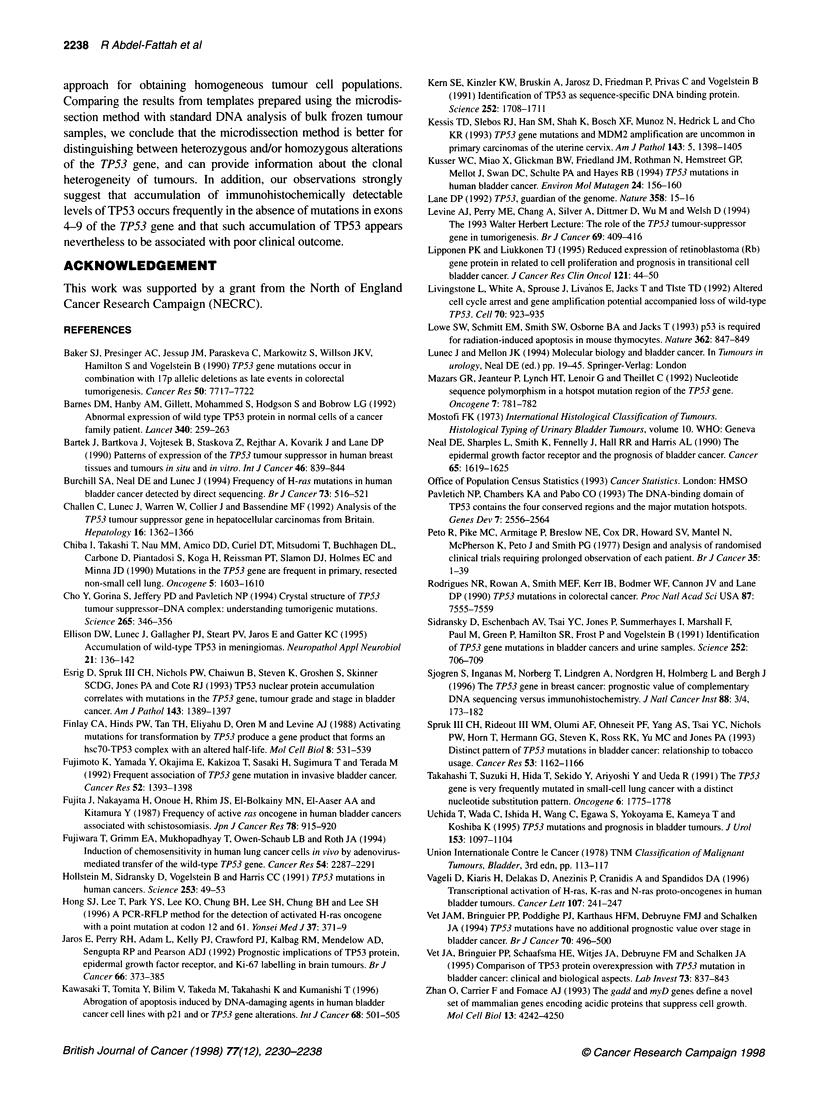# Alterations of TP53 in microdissected transitional cell carcinoma of the human urinary bladder: high frequency of TP53 accumulation in the absence of detected mutations is associated with poor prognosis.

**DOI:** 10.1038/bjc.1998.371

**Published:** 1998-06

**Authors:** R. Abdel-Fattah, C. Challen, T. R. Griffiths, M. C. Robinson, D. E. Neal, J. Lunec

**Affiliations:** Cancer Research Unit, The Medical School, University of Newcastle upon Tyne, UK.

## Abstract

**Images:**


British Journal of Cancer (1998) 77(12), 2230–2238
© 1998 Cancer Research Campaign

### Alterations of *TP53* in microdissected transitional cell carcinoma of the human urinary bladder: high frequency of TP53 accumulation in the absence of detected mutations is associated with poor prognosis

**R Abdel-Fattah[1], C Challen[1], TRL Griffiths[2], MC Robinson[3], DE Neal[2,3] and J Lunec[1]**

[1]Cancer Research Unit and [2]Department of Surgery, The Medical School, University of Newcastle upon Tyne, Framlington Place, Newcastle upon Tyne NE2 4HH; [3]Department of Pathology, Freeman Hospital, Freeman Rd, Newcastle upon Tyne NE7 7DN, UK

**Summary** We have used microdissection of paraffin-embedded histological sections and polymerase chain reaction (PCR)-based direct DNA sequencing for 54 transitional cell carcinoma (TCC) of the bladder, to examine critically the association between TP53 nuclear accumulation determined by immunohistochemistry and the presence of *TP53* mutations, and to examine their relationship to tumour stage and grade, as well as patient survival. There was a significant association between the presence of TP53-positive nuclei (> 10%) and a higher histological stage and grade ($P$ = 0.0115, $P$ = 0.0151 respectively; Fisher's exact). A significant association between *TP53* gene mutations and TP53 nuclear reactivity in more than 10% of tumour cell nuclei was also observed ($P$ = 0.0003; Fisher's exact). Mutations were detected in 18/54 (33%) cases together with the wild-type sequence when analysed from bulk frozen samples, with significant clustering of mutations in exons 7 and 8. The microdissection method distinguished more clearly between heterozygous and/or homozygous alterations of the *TP53* tumour-suppressor gene, and clearly showed frequent accumulation of TP53 in the absence of mutations. When microdissecting immunonegative regions from the same paraffin sections, three out of ten samples showed the identical mutations detected in the immunopositive regions. There was a significant association between TP53 immunoreactivity in more than 50% of tumour cell nuclei and decreased survival among all patients ($P$ = 0.0325; log-rank test). The patients with *TP53* mutations showed a trend for a shorter survival period; however, the association was not statistically significant at the 95% confidence level ($P$ = 0.132; log-rank test). In conclusion, our observations show that accumulation of TP53 occurs frequently in the absence of mutations, and that such accumulation is nevertheless associated with poor survival when it occurs in a high proportion (> 50%) of tumour cell nuclei.

**Keywords:** *TP53*; mutation; immunohistochemistry; bladder cancer; microdissection

Ten thousand individuals per year in England and Wales develop bladder cancer and 5800 die as a result of the disease (Office of Population Census Statistics, 1993). A total of 90% of bladder tumours are transitional cell carcinoma (TCC), whereas less common histological types include squamous cell carcinoma, adenocarcinoma and sarcoma. Molecular genetic and immunopathological analyses of bladder cancer have identified abnormalities in a number of chromosomes and genes that appear to be implicated in the development and progression of such tumours. These include activation of the H-*RAS* oncogene (Fujita et al, 1987; Burchill et al, 1994; Hong et al, 1996; Vageli et al, 1996), increased expression of epidermal growth factor receptor (Neal et al, 1990) and inactivation of the retinoblastoma gene (Lipponen and Liukkonen, 1995) as well as frequent abnormalities of the *TP53* tumour-suppressor gene.

*TP53* is a key gene in carcinogenesis, being involved in cell cycle control and preservation of genomic integrity. It co-ordinates the cellular response to DNA damage and other cellular stresses by inducing cell cycle arrest (Livingstone et al, 1992) or apoptosis (Lowe et al, 1993; Fujiwara et al, 1994), depending on the severity of damage and cell type. TP53 can bind to specific DNA sequences and activate the expression of genes containing TP53-dependent promoter regions (Kern et al, 1991; Zhan et al, 1993). A number of genes have been identified that can be induced in response to *TP53* expression, such as *MDM2*, *WAF1*, *GADD45* and *BAX*, which have been shown to control or mediate some of the downstream functions of *TP53*. Loss of *TP53* function, most commonly through point mutation within one of the evolutionarily conserved domains, occurs in approximately half of most major cancers (Baker et al, 1990; Lane 1992), including bladder tumours (Sidransky et al, 1991; Kusser et al, 1994; Vet et al, 1995; Kawasaki et al, 1996).

Many of the point mutations of the *TP53* gene lead to accumulation of a stabilized protein product that is detectable by immunohistochemistry (Finlay et al, 1988). Our initial sequencing studies using bulk tumour DNA extracts indicated that only approximately 50% of positively staining samples show evidence of *TP53* mutations when analysed by PCR-based direct DNA sequencing. Because of tumour heterogeneity and the presence of normal stromal cells, wild-type *TP53* can mask the detection of small amounts of mutated *TP53*. It is therefore difficult to conclude from such studies that accumulation of immunohistochemically detectable TP53 can occur in the absence of mutations. Thus, the aim of this study was to examine more critically the direct association between TP53 protein overexpression and the presence of

*Received 4 August 1997*
*Revised 18 November 1997*
*Accepted 5 December 1997*

*Correspondence to:* J Lunec mutations and to compare the relationship of these observations to patient survival. This was performed by microdissecting the immunopositive regions from paraffin-embedded sections for selective molecular analysis. The results of this were compared with those obtained from microdissection of the immunonegative regions from the same histological sections and, in addition, the results were compared with molecular analysis performed on bulk frozen tumours from the same patients.

### MATERIALS AND METHODS

### Tumour samples

A total of 54 TCC of the bladder were available for study from the Urology Department of the Freeman Hospital, Newcastle Upon Tyne, UK. Parts of the tumours were snap frozen in liquid nitrogen for subsequent PCR and sequencing. The remaining parts were fixed in formalin, paraffin embedded and sectioned for routine histology, immunohistochemistry, microdissection, PCR and sequencing. Tumours were graded according to World Health Organization guidelines (Mostofi, 1973), and staged according to the TNM classification (UICC, 1978).

### Immunohistochemistry

Paraffin-embedded sections (4 μm) were incubated with the DO-7 monoclonal antibody (DO-7; Novocastra) to the TP53 protein at a 1:250 dilution using standard immunohistochemical staining methods, as described previously (Jaros et al, 1992). Sections of colorectal carcinoma previously found to show intense nuclear staining for DO-7 were used as positive controls. Negative controls for background staining were performed by omitting the primary antibody in each case.

### Grading and assessment of immunostaining

Slides were examined for the extent and intensity of nuclear staining in areas of tumour and for background staining. Representative sections of each block were stained with haematoxylin and eosin and reviewed by a pathologist (MC Robinson) to check previous grading. The staining was assessed by scoring 1000 cells in the area of highest positivity, assessed independently by two observers using a light microscope (×400). The extent of nuclear reactivity was scored as the percentage of positively staining tumour cell nuclei.

### Microdissection and DNA extraction

TP53-positive immunoreactive cells were specifically marked on the stained 4-μm paraffin-embedded sections. Adjacent unstained and unheated 18 μm sections were marked on the same TP53-immunopositive regions. The circumscribed tumour regions were separated from adjacent tissues by direct scraping of the slides using a sterile needle under the light dissecting microscope. The microdissected tissues were then placed in sterile microfuge PCR tubes. Previous DNA extraction protocols for paraffin-embedded tissue have used xylene to dissolve the paraffin wax, and then ethanol to remove the xylene, followed by rehydration through different percentages of alcohol, and usually take more than 2 h. Therefore, in order to save time, a more rapid method was developed in which the microdissected sections were directly resuspended in 100–150 μl of digestion buffer (50 mM Tris at pH 8.5, 1 mM EDTA and 0.5% Tween 20), and heated at 95°C in the thermal cycler for 1 min. The paraffin wax was then removed from the supernatant using a sterile needle. Proteinase K was added to the buffer to a final concentration of 20 μg $ml^{-1}$, and the samples were incubated overnight at 37°C. The proteinase K was then heat inactivated at 95°C for 10 min, and the samples stored frozen at –20°C before aliquots were taken for direct PCR amplification as required. To compare between TP53-immunopositive and -immunonegative regions, microdissection of the immunonegative regions from the same histological sections was also performed. The samples were also analysed for *TP53* mutations using bulk frozen tumours without microdissection. DNA extraction from frozen tumours was performed as previously described (Burchill et al, 1994).

### PCR amplification and direct sequencing of exons 4–9 of the *TP53* gene

The method of PCR amplification and direct sequencing of exons 4–9 of the *TP53* gene from genomic DNA, using the biotin method for purification of a single-stranded template, and the sequences of the primer pairs used, have previously been described in detail (Challen et al, 1992; Ellison et al, 1995). All PCR reactions were carefully controlled to ensure absence of background products when a DNA sample was not included. Plastic tubes, tips and solutions (excluding primers) were pretreated with UV irradiation to ensure destruction of any contaminating DNA template.

### Statistical analysis

Association between *TP53* sequencing and TP53 immunoexpression were assessed by Fisher's exact test (two tailed). In all tests $P < 0.05$ was considered to be statistically significant according to the usual convention. The Kaplan–Meier method (GraphPad Prism package) was used to estimate survival probability as a function of time, and the log-rank test to examine differences in survival between subgroups (Peto et al, 1977). Multivariate analysis was performed by the Cox proportional hazards method using the SPSS statistical software package.

### RESULTS

### Immunohistochemical analysis

Staining for TP53 was predominantly nuclear (Figure 1A). The extent of positive staining of the tumour nuclei for TP53 varied considerably. In 12/54 (22%) sections, there was no detectable immunoreactivity. The number of cases in different staining categories, based on percentage of tumour nuclei showing positive immunoreactivity, is shown in Table 1. There was a considerable heterogeneity in the staining pattern from tumour to tumour. Whereas most tumours (62%) showed a diffuse intense pattern of nuclear staining (> 75% positivity) (Figure 1B), others (38%) showed staining of individual cell nuclei or small groups of cells only. There was a statistically significant association between diffuse staining and a higher histological stage ($P = 0.0006$; Fisher's exact); 23/35 (66%) T2–T4 tumours and 3/19 (16%) Ta–T1 tumours showed the diffuse staining pattern. Twelve tumours showed no detectable TP53 immunoreactivity, and six (50%) of these were of muscle-invasive stage T3. There was a statistically significant association between TP53-positive nuclei

© Cancer Research Campaign 1998

A

B

**Figure 1** Immunohistochemical staining with DO7 monoclonal antibody. **A** Intense nuclear staining. **B** greater than 75% TP53 nuclear reactivity (diffuse pattern)

(> 10%) and a higher histological stage; 9/19 (47%) tumours with lower stage (Ta–T1) and 29/35 (83%) tumours with higher stage (T2–T4) showed TP53 reactivity in more than 10% of tumour cell nuclei ($P$ = 0.0115; Fisher's exact). There was also a significant association between TP53-positive nuclei (> 10%) and a higher histological grade; 10/21 (48%) tumours with lower grade (I and II) and 27/33 (82%) tumours with grade III showed TP53 reactivity in more than 10% of tumour cell nuclei ($P$ = 0.0151; Fisher's exact). Only the percentage of positive cells was taken into account for statistical analysis, regardless of any variation in intensity.

### Molecular analysis of *TP53* from bulk frozen samples

Full sequence analysis was carried out for exons 4–9 of the *TP53* gene, covering the highly conserved domains. Table 2 shows the mutations detected in 18/54 samples analysed. The mutations were predominantly of point missense type. The incidence of *TP53* mutations was higher in muscle-invasive tumours [14/35 (40%)] compared with superficial tumours [4/19 (21%)]; however, the association was not statistically significant ($P$ = 0.2293; Fisher's exact). Similarly, the incidence of *TP53* mutations was greater in higher grade (III) tumours [14/33 (42%)] than lower grade (I and II)

© Cancer Research Campaign 1998

**Table 1** Histological classification of transitional cell carcinoma, TP53 nuclear reactivity using the DO-7 monoclonal antibody and patient follow-up

| | Total | % TP53 nuclear reactivity | | | | | Follow-up (months) | |
|---|---|---|---|---|---|---|---|---|
| | | 0 | 0–10 | 10–50 | 50–75 | >75 | Median | Range |
| Grade | | | | | | | | |
| I | 1 | 1 | 0 | 0 | 0 | 0 | 74 | 74–74 |
| II | 20 | 5 | 5 | 3 | 3 | 4 | 30 | 2–77 |
| III | 33 | 6 | 0 | 3 | 2 | 22 | 19 | 1–84 |
| Stage | | | | | | | | |
| Ta | 9 | 5 | 2 | 0 | 0 | 2 | 54 | 9–75 |
| T1 | 10 | 1 | 2 | 3 | 3 | 1 | 23 | 2–75 |
| T2 | 6 | 0 | 1 | 2 | 0 | 3 | 75 | 9–84 |
| T3 | 22 | 6 | 0 | 1 | 1 | 14 | 16 | 2–80 |
| T4 | 7 | 0 | 0 | 1 | 0 | 6 | 9 | 1–36 |

T, stage (Ta–T1, superficial; T2–T4, muscle invasive). G, grade (I, well differentiated; II, moderately differentiated; III, undifferentiated).

**Table 2** *TP53* mutations found in 18/54 transitional cell carcinoma of the bladder analysed by direct DNA sequencing

| Patient number | Stage | Grade | Exon | Codon | Base change | Amino acid change |
|---|---|---|---|---|---|---|
| 1 | Ta | II | 8 | 287 | GAG–TAG | Glu–Stop[b] |
| 10 | T1 | III | 6 | 192 | CAG–TAG | Gln–Stop[b] |
| 11 | T1 | II | 8 | 271 | GAG–AAG | Glu–Lys |
| 12 | T1 | II | 5 | 155 | ACC–AGC | Thr–Ser |
| 20 | T2 | III | 7 | 248 | CGG–CAG | Arg–Gln |
| 21 | T2 | III | 7 | 234 | TAC–TGC | Tyr–Cys |
| 22 | T2 | III | 8 | 285 | GAG–TAG | Glu–Stop[b] |
| 30 | T3b | III | 8 | 282 | CGG–TGG | Arg–Trp |
| 37 | T3b | III | 6 | 224 | GAG–AAG | Glu–Lys |
| 38 | T3a | III | 7 | 256 | ACA–GCA | Thr–Ala |
| 42 | T3b | III | 7 | 248 | CGG–TGG | Arg–Trp |
| 44 | T3b | III | 7 | 245 | GGC–AGC | Gly–Ser |
| 45 | T3a | III | 8 | 272 | GTG–ATG | Val–Met |
| | | | 6 | 213[a] | CGA–CGG | Arg–Arg |
| 48 | T4a | III | 8 | 275 | TGT–TAT | Arg–Tyr |
| 49 | T4b | II | 7 | 248 | CGG–CTG | Arg–Leu |
| 50 | T4b | III | 7 | 241 | TCC–TTC | Ser–Phe |
| 51 | T4b | III | 8 | 277 | TGT–TAT | Cys–Tyr |
| 53 | T4a | III | 7 | 235 | AAC–AGC | Asn–Ser |

[a]Polymorphism. [b]Nonsense mutation.

tumours [4/21 (19%)], but again this difference was not statistically significant ($P = 0.1376$; Fisher's exact). Of 18 mutations, 14 (78%) were transitions and 4/18 (22%) were transversions. Although all samples with *TP53* mutations showed reactivity in more than 30% of tumour cell nuclei, 17/36 (47%) samples that did not have *TP53* mutations in exons 4–9 showed reactivity in more than 30% of tumour cell nuclei detected by immunohistochemistry, and 12 of these 17 (71%) were of high stage (T2–T4). There was an extremely significant association between the presence of *TP53* mutations and TP53 immunoreactivity in more than 10% of tumour cell nuclei ($P = 0.0003$; Fisher's exact), with most of the cases of mutations [16/18; (89%)] occurring in the strongly staining group (>75% positive nuclei) (Figure 2).

The *TP53* mutations detected were single nucleotide changes in exons 5, 6, 7 and 8, which were predominantly point missense mutations resulting in a predicted amino acid substitution (Table 2). No mutations in exons 4 or 9 were found. A striking feature was that the mutations showed evidence of clustering in exons 7 and 8 of the *TP53* gene. An overall comparison of the incidence of mutations reported in the literature, with respect to the distribution of mutations along the *TP53* gene in bladder cancer, is summarized in Table 3 and Figure 3. Three samples (nos 1, 10 and 22)

**Table 3** Proportion of TP53 mutations found in TCC of the bladder in exons 4–9 of the TP53 gene compiled from five separate studies

| *TP53* exons | 4 | 5 | 6 | 7 | 8 | 9 |
|---|---|---|---|---|---|---|
| Sidransky et al (1991) | ND | 2 | 2 | 5 | 2 | 0 |
| Fujimoto et al (1992) | 2 | 2 | 0 | 0 | 2 | 1 |
| Esrig et al (1993) | ND | 6 | 3 | 8 | 15 | ND |
| Spruck III et al (1993) | ND | 7 | 4 | 7 | 19 | ND |
| Vet et al (1994) | ND | 3 | 1 | 1 | 3 | ND |
| Uchida et al (1995) | 2 | 6 | 1 | 1 | 10 | 0 |
| Newcastle | 0 | 1 | 2 | 8 | 7 | 0 |
| Total | 4 | 27 | 13 | 30 | 58 | 1 |
| Proportion | 4% | 27% | 13% | 30% | 58% | 1% |

ND, not done.

**Figure 2** Scatter plot of percentage nuclear staining for TP53 in mutant ($n$ = 18) compared with wild-type subgroups ($n$ = 36)

**Figure 3** Distribution of mutations along the *TP53* gene in transitional cell carcinoma of the bladder harboured nonsense mutations in exon 6 (codon 192) and exon 8 (codons 287 and 285), which were still associated with positive staining by immunohistochemistry with the DO-7 monoclonal antibody that recognizes an epitope localized to codons 35 to 45 (Table 1). Only one sample (no. 45) harboured multiple sequence alterations, one of which was a silent heterozygous alteration in exon 6 at codon 213, involving an A to G nucleotide substitution (Figure 4A). This polymorphism has been described previously (Mazars et al, 1992; Kessis et al, 1993; Vet et al, 1994). The other mutation in the same sample was located in exon 8 at Val-272; a well-known hot spot for mutation of the *TP53* gene and shown in Figure 5 as an additional sequencing example. Three mutations were observed at Arg-248 of exon 7, each involving different base pair changes, and the mutations in exon 8 were all clustered in the region between codon 271 and codon 287 (Table 2). All mutations appeared to be heterozygous from bulk sample analysis.

### Molecular analysis of the *TP53* gene from microdissected TP53-immunopositive and -immunonegative regions of paraffin-embedded sections

A panel of 23 paraffin-embedded sections from the 54 samples was selected for microdissection. The samples were divided into two groups: group 1 consisted of 10 samples that had shown p53 mutations when analysed from the bulk frozen tumours; group 2 consisted of ten (T2–T4) muscle-invasive tumours and three (Ta–T1) superficial tumours that had not shown mutations but were positive for p53 nuclear reactivity. TP53-immunopositive regions as well as -immunonegative regions were microdissected in the first group of samples to test whether immunonegative regions represented subclones of tumour cells without mutations.

Upon microdissection of TP53-immunopositive regions, all mutations were confirmed in the first group of samples. However, the microdissection method also showed there to be an unequivocal homozygous pattern of mutated *TP53* in two cases (no. 45 and

**Figure 4** Sequencing gels for sample no. 45. **A** An apparent heterozygous polymorphism at C213 detected from sequencing frozen tumour without microdissection. **B** The same polymorphism in the same sample but in a homozygous pattern, showing evidence of allele loss, detected from sequencing a microdissected paraffin section. **C** Wild-type sequence from normal bladder sample

 © Cancer Research Campaign 1998

**Figure 5** Sequencing gel for sample no. 45 showing a mutation in exon 8 at C272 (G:C to A:T)

no. 37). Without microdissection, it was impossible to distinguish homozygous mutations from heterozygous mutations because of normal cell contamination or tumour cell population heterogeneity. When microdissecting immunonegative regions, three out of ten showed the identical previously found mutations, suggesting that immunohistochemistry may give false-negative results for the detection of mutations in some circumstances. No new mutations were found on microdissection. The full results of the microdissection analysis are summarized in Figure 6.

### TP53 immunoreactivity, *TP53* mutation and survival

The relationship between survival and *TP53* status for 52 patients is shown in the Kaplan–Meier survival curves (Figure 7). The maximum patient follow-up time was 84 months and the median follow-up time was 23 months. There was a significant association between TP53 immunoreactivity in more than 50% of tumour cell nuclei and decreased survival among all patients ($\chi^2$ = 4.571; $P$ = 0.0325, log-rank test) (Figure 7A). The patients with *TP53* mutations showed a trend towards a shorter survival period; however, this association was not statistically significant when considered in isolation ($\chi^2$ = 2.258; $P$ = 0.1329, log-rank test) (Figure 7B). However, when the group with no evidence of mutations was subdivided into subgroups of high positive staining (>50% positive nuclei) and low positive staining (>50% positive nuclei), the mutant group was not distinguishable from the high positive-staining non-mutant group. Furthermore, there was a significant trend towards better survival for the low positive-staining non-mutant subgroup ($P$ = 0.0371; log-rank test for trend; Figure 7C). In multivariate analysis using a Cox proportional hazards model, including sex, stage, age, grade, *TP53* mutational status and DO-7 staining, with stepwise conditional removal or addition of variables, only stage was found to be significantly related to survival ($P$ = 0.009).

### DISCUSSION

*TP53* mutation is a common genetic alteration in many human malignancies. A wide range of mutations stabilize the TP53 protein, and its consequent accumulation is detectable by immunohistochemistry. Although initial studies indicated an association

**Figure 6** A summary of immunohistochemistry and sequencing results obtained from frozen tumours and microdissection of immunopositive and -negative regions from paraffin-embedded sections in 54 transitional cell carcinoma of the bladder

**Figure 7** Kaplan–Meier analysis of survival in 52 patients with transitional cell carcinoma of the bladder. **A** TP53 nuclear expression in >50% of tumour nuclei was the most significant variable associated with survival (- - -, TP53 immunoreactivity <50%, $n$ = 23; —, TP53 immunoreactivity >50%, $n$ = 29). Patients with TP53 nuclear overexpression were twice as likely to die of bladder cancer than those whose tumours had minimal or no TP53 overexpression ($\chi^2$ = 4.571, $P$ = 0.0325). **B** The patients with *TP53* mutations showed a trend towards shorter survival time (- - -), wild-type, $n$ = 34; —, mutant, $n$ = 18), but the association was not significant ($\chi^2$ = 2.258, $P$ = 0.1329). **C** A comparison between mutant, – • –, wild type with > 50% positively staining nuclei (—) and wild type with < 50% positively staining nuclei (- - -). $\chi^2$ = 4.345, $P$ = 0.0371

between immunohistochemically detectable levels of protein and the presence of mutations (Bartek et al, 1990; Rodrigues et al, 1990), this relationship has not previously been critically examined by microdissection studies.

Fifty-four TCC of the bladder were analysed by immunohistochemistry for TP53 nuclear accumulation. Whereas there was significant association between TP53 immunohistochemistry and mutation detected from frozen samples ($P$ = 0.0003; Fisher's exact), a significant proportion of cases showed high frequency of TP53 immunoreactivity in the absence of detectable mutations (Figure 2). Of 36 samples, 17 (47%) that did not have *TP53* mutations in exons 4–9 showed reactivity in more than 30% of tumour cell nuclei detected by immunohistochemistry, and 12 of these 17 (71%) were muscle-invasive (T2–T4) tumours. If the proportion of tumour cells containing *TP53* mutations is relatively low compared with normal cells or tumour cells containing wild-type *TP53*, molecular analysis may not detect the mutations in the tumour cells. Our previous mixing studies with mutant and wild-type DNA have shown that detection of mutations by direct sequencing is lost if the proportion of mutant DNA falls below 25% (Burchill et al, 1994). When 10/12 muscle-invasive tumours, which had shown no mutations from sequencing bulk frozen tumours, were examined by microdissection and direct sequencing of only the immunopositive regions from paraffin-embedded sections, these microdissected regions still proved to be of normal wild-type *TP53* sequence. Several investigators (Barnes et al, 1992; Sjogren et al, 1996) have reported discrepancies between TP53 protein expression and mutation status. It is known that a range of cellular stresses or the action of different combinations of activated oncogenes or tumour-suppressor gene alterations can influence wild-type TP53 accumulation (Lane, 1992).

Although most *TP53* mutations in diverse types of cancer have been found in exons 4–9 (Hollstein et al, 1991), it is possible that mutations exist outside these regions of the gene; however, such mutations have been described in less than 8% of tumours from many varied studies (Levine et al, 1994) and could not account for the absence of mutations in approximately half of the samples with a high frequency (>50%) of positive nuclear staining for TP53 (Figure 2).

In a separate aspect of our study we also examined a panel of ten tumour sections that were analysed previously by PCR-based sequencing from frozen tumours and had shown *TP53* mutations. Both TP53-immunopositive and -immunonegative regions from the ten paraffin sections were microdissected. In all cases mutations were confirmed from microdissecting the immunopositive regions. However, unlike the prior frozen bulk sample analysis, the microdissection method gave an unequivocal homozygous pattern of mutated *TP53* in two cases. The sample (no. 45) that had shown an apparent heterozygous polymorphism at C213, when DNA extracted from frozen tumour without microdissection was analysed, revealed the presence of the same polymorphism but in a homozygous pattern on microdissection (Figure 4B). Another sample (no. 37) that had previously shown evidence of both wild-type and mutant sequence from bulk sample analysis turned out to have a homozygous mutation at C224 when analysed by microdissection. This suggests that microdissection is more reliable for distinguishing between heterozygous and homozygous mutations. The technique is simple, rapid, highly selective and at little additional cost improves the accuracy of direct sequencing for the detection of gene alterations specifically in tumour cells or associated with altered immunohistochemical staining.

 © Cancer Research Campaign 1998

Microdissection also has the potential to shed light on the clonal evolution of the tumours and their malignant progression. Interestingly, the sample (no. 45) that has shown the homozygous polymorphism at C213 also had an apparent heterozygous mutation at Val-272 confirmed by microdissection in the same DNA sample (Figure 5). This suggests that deletion of one *TP53* allele occurred before a subsequent mutation, which is only present in a subpopulation of the tumour cells, or alternatively there could have been a duplication of chromosome 17 following initial allele loss and subsequently a mutation occurring in one of these alleles. The latter possibility would be more consistent with the microdissected population including only cells staining positively for TP53.

Microdissection of heterogeneously staining sections also revealed, in a proportion of cases, that mutations can be present despite absence of TP53 staining, even though adjacent areas with the same mutation show positive staining. When immunonegative regions were microdissected from the ten samples that had shown *TP53* mutations, three out of ten showed the same mutations, indicating that immunohistochemistry may give false-negative results. Technical artefacts could explain the absence of staining in such cases. Alternatively these regions may genuinely represent areas of the tumour in which the mutant form does not accumulate. It is possible that the presence of mutations alone is not the sole determinant of protein accumulation. This is evident with Li–Fraumeni patients, who do not show evidence of positive immunohistochemical staining of their normal tissues in which all cells are heterozygous for *TP53* mutations.

Our data support previous findings in bladder cancer that have shown that *TP53* mutations are more prevalent in high-grade invasive bladder cancer (Fujimoto et al, 1992; Vet et al, 1994; Kusser et al, 1994). We analysed 54 tumour samples and 18 mutations were detected, 14 of which were grade III invasive tumours. The increased frequency of *TP53* mutations in late-stage high-grade tumours suggests that *TP53* mutations are involved in disease progression. The detection of *TP53* mutations in four superficial (Ta–T1) low-grade tumours indicates that *TP53* aberrations may be an early indicator of subsequent invasive progression in bladder cancer and suggests that they may be involved in the evolution of the tumour to a more malignant form. This is consistent with observations showing that loss of *TP53* function leads to genetic instability and hence the acceleration of the progression of the cells along the pathway towards invasive metastatic disease.

A marked clustering of mutations in exons 7 and 8 was evident, corresponding to conserved domains IV and V of the *TP53* gene, in keeping with other published studies (Table 3), although not previously noted in the literature. A total of 15/18 mutations (83%) were located in exons 7 and 8 (Table 2). This corresponds to a region of TP53 that is involved in DNA binding (Pavletich et al, 1993). Three samples showed mutations at Arg-248 of exon 7, which is one of the most frequently altered sites in *TP53*. Arginine is coded for by CGN and hence is a site for spontaneous mutations because of methylation-induced deamination of 5′-methylcytosine, and this is observed in sample 42 (Table 2). In addition, Arg-248 interacts with the DNA minor groove in the A-T-rich region of the consensus DNA-binding sequence [PuPuPuC(A/T)-(T/A)GPyPyPy] (Cho et al, 1994). Thus, Arg-248 performs the critical job of anchoring *TP53* to the DNA minor groove, and missense mutations at this point within the DNA binding domain of *TP53* correlate with loss of DNA binding capacity, resulting in loss of *TP53* suppressor function.

In addition, among the other frequently mutated residues, two hot spots stood out: Gly-245 and Arg-282 (Table 2). Arg-282 from the $H_2$ alpha helix plays a structural role in the loop–sheet–helix motif, being involved in the packing of the $H_2$ helix against the $\beta$ hairpin and the L1 loop (Cho et al, 1994). On the other hand, Gly-245 plays a critical role in the formation of two hydrogen bonds, one with the backbone carbonyl of Cys-247 (one of the zinc ligands), and the other with the guanidinium group of Arg-249 (another hot spot mutation). Thus, these two hot spot residues also appear to play a critical role in stabilizing the structure of the DNA-binding surface of *TP53* (Cho et al, 1994).

Analysis of the frequency, type and site of *TP53* mutations can give important clues to aetiological factors and assist efforts to distinguish the tumours associated with a particular carcinogen exposure. The frequency and type of *TP53* gene mutations detected vary with cancer type, indicative of the involvement of different aetiological factors and environmental carcinogens and the role of *TP53* in particular tissues. For example, in lung cancer, G:C to T:A transversions are often observed and such mutations have been shown to be induced by benzo(a)pyrene and polycyclic aromatic hydrocarbons, which are implicated as carcinogens in cigarette smoking (Chiba et al, 1990; Takahashi et al, 1991). In the present study, 14/18 (78%) mutations were transitions and 4/18 (22%) were transversions. The proportion of transitions was not significantly different from that expected for spontaneous mutations (Lunec and Mellon, 1994). Even though smoking is implicated as a risk factor in bladder cancer, this is different to the pattern seen in lung cancer, for which there is an excess of transversions. This is because benz(o) pyrene and polycyclic aromatic hydrocarbons from cigarette smoke react locally in the lung and do not get into the circulation in significant quantities because of their lack of aqueous solubility. Alkylating agents such as nitrosamines are more likely carcinogens in the circulation, which arise through tobacco smoke inhalation, and these are more likely to be associated with transitions, as found in our study.

A large body of evidence has indicated that *TP53* is not only important in the development and progression of cancer, but is also a major determinant of response to chemotherapy and radiotherapy. Although there was a trend for better survival in the group with no *TP53* mutation (Figure 7B), this did not achieve statistical significance ($P = 0.132$; log-rank test). TP53 nuclear accumulation in more than 50% of tumour cells was the most significant variable associated with survival when considered alone ($P = 0.0325$; log-rank test). Patients with TP53 nuclear accumulation were twice as likely to die of bladder cancer than patients whose tumours had minimal or no immunohistochemically detectable accumulation. This implies that accumulation of wild-type TP53 is associated with poor prognosis. Of further note, when the group of cases showing absence of mutations was subdivided into high- (>50%) and low (<50%)-frequency TP53-staining groups and compared against the mutant group, a statistically significant trend was evident. This showed that the group with no detectable mutations and low-frequency TP53 staining had the best long-term survival rate, whereas the group with no mutations, but with high-frequency accumulation of wild-type TP53 did poorly and was indistinguishable from the mutant group taken as a whole (Figure 7C). This has not been previously noted and raises questions about the functional status of the accumulated TP53 in non-mutant cases, which should be explored further.

In conclusion, the direct selection of immunopositive cells from histological sections using microdissection is a more reliable

© Cancer Research Campaign 1998 

approach for obtaining homogeneous tumour cell populations. Comparing the results from templates prepared using the microdissection method with standard DNA analysis of bulk frozen tumour samples, we conclude that the microdissection method is better for distinguishing between heterozygous and/or homozygous alterations of the *TP53* gene, and can provide information about the clonal heterogeneity of tumours. In addition, our observations strongly suggest that accumulation of immunohistochemically detectable levels of TP53 occurs frequently in the absence of mutations in exons 4–9 of the *TP53* gene and that such accumulation of TP53 appears nevertheless to be associated with poor clinical outcome.

### ACKNOWLEDGEMENT

This work was supported by a grant from the North of England Cancer Research Campaign (NECRC).

### REFERENCES

Baker SJ, Presinger AC, Jessup JM, Paraskeva C, Markowitz S, Willson JKV, Hamilton S and Vogelstein B (1990) *TP53* gene mutations occur in combination with 17p allelic deletions as late events in colorectal tumorigenesis. *Cancer Res* **50**: 7717–7722

Barnes DM, Hanby AM, Gillett, Mohammed S, Hodgson S and Bobrow LG (1992) Abnormal expression of wild type TP53 protein in normal cells of a cancer family patient. *Lancet* **340**: 259–263

Bartek J, Bartkova J, Vojtesek B, Staskova Z, Rejthar A, Kovarik J and Lane DP (1990) Patterns of expression of the *TP53* tumour suppressor in human breast tissues and tumours *in situ* and *in vitro*. *Int J Cancer* **46**: 839–844

Burchill SA, Neal DE and Lunec J (1994) Frequency of H-*ras* mutations in human bladder cancer detected by direct sequencing. *Br J Cancer* **73**: 516–521

Challen C, Lunec J, Warren W, Collier J and Bassendine MF (1992) Analysis of the *TP53* tumour suppressor gene in hepatocellular carcinomas from Britain. *Hepatology* **16**: 1362–1366

Chiba I, Takashi T, Nau MM, Amico DD, Curiel DT, Mitsudomi T, Buchhagen DL, Carbone D, Piantadosi S, Koga H, Reissman PT, Slamon DJ, Holmes EC and Minna JD (1990) Mutations in the *TP53* gene are frequent in primary, resected non-small cell lung. *Oncogene* **5**: 1603–1610

Cho Y, Gorina S, Jeffery PD and Pavletich NP (1994) Crystal structure of *TP53* tumour suppressor–DNA complex: understanding tumorigenic mutations. *Science* **265**: 346–356

Ellison DW, Lunec J, Gallagher PJ, Steart PV, Jaros E and Gatter KC (1995) Accumulation of wild-type TP53 in meningiomas. *Neuropathol Appl Neurobiol* **21**: 136–142

Esrig D, Spruk III CH, Nichols PW, Chaiwun B, Steven K, Groshen S, Skinner SCDG, Jones PA and Cote RJ (1993) TP53 nuclear protein accumulation correlates with mutations in the *TP53* gene, tumour grade and stage in bladder cancer. *Am J Pathol* **143**: 1389–1397

Finlay CA, Hinds PW, Tan TH, Eliyahu D, Oren M and Levine AJ (1988) Activating mutations for transformation by *TP53* produce a gene product that forms an hsc70-TP53 complex with an altered half-life. *Mol Cell Biol* **8**: 531–539

Fujimoto K, Yamada Y, Okajima E, Kakizoa T, Sasaki H, Sugimura T and Terada M (1992) Frequent association of *TP53* gene mutation in invasive bladder cancer. *Cancer Res* **52**: 1393–1398

Fujita J, Nakayama H, Onoue H, Rhim JS, El-Bolkainy MN, El-Aaser AA and Kitamura Y (1987) Frequency of active *ras* oncogene in human bladder cancers associated with schistosomiasis. *Jpn J Cancer Res* **78**: 915–920

Fujiwara T, Grimm EA, Mukhopadhyay T, Owen-Schaub LB and Roth JA (1994) Induction of chemosensitivity in human lung cancer cells *in vivo* by adenovirus-mediated transfer of the wild-type *TP53* gene. *Cancer Res* **54**: 2287–2291

Hollstein M, Sidransky D, Vogelstein B and Harris CC (1991) *TP53* mutations in human cancers. *Science* **253**: 49–53

Hong SJ, Lee T, Park YS, Lee KO, Chung BH, Lee SH, Chung BH and Lee SH (1996) A PCR-RFLP method for the detection of activated H-ras oncogene with a point mutation at codon 12 and 61. *Yonsei Med J* **37**: 371–9

Jaros E, Perry RH, Adam L, Kelly PJ, Crawford PJ, Kalbag RM, Mendelow AD, Sengupta RP and Pearson ADJ (1992) Prognostic implications of TP53 protein, epidermal growth factor receptor, and Ki-67 labelling in brain tumours. *Br J Cancer* **66**: 373–385

Kawasaki T, Tomita Y, Bilim V, Takeda M, Takahashi K and Kumanishi T (1996) Abrogation of apoptosis induced by DNA-damaging agents in human bladder cancer cell lines with p21 and or *TP53* gene alterations. *Int J Cancer* **68**: 501–505

Kern SE, Kinzler KW, Bruskin A, Jarosz D, Friedman P, Privas C and Vogelstein B (1991) Identification of TP53 as sequence-specific DNA binding protein. *Science* **252**: 1708–1711

Kessis TD, Slebos RJ, Han SM, Shah K, Bosch XF, Munoz N, Hedrick L and Cho KR (1993) *TP53* gene mutations and MDM2 amplification are uncommon in primary carcinomas of the uterine cervix. *Am J Pathol* **143**: 5, 1398–1405

Kusser WC, Miao X, Glickman BW, Friedland JM, Rothman N, Hemstreet GP, Mellot J, Swan DC, Schulte PA and Hayes RB (1994) *TP53* mutations in human bladder cancer. *Environ Mol Mutagen* **24**: 156–160

Lane DP (1992) *TP53*, guardian of the genome. *Nature* **358**: 15–16

Levine AJ, Perry ME, Chang A, Silver A, Dittmer D, Wu M and Welsh D (1994) The 1993 Walter Herbert Lecture: The role of the *TP53* tumour-suppressor gene in tumorigenesis. *Br J Cancer* **69**: 409–416

Lipponen PK and Liukkonen TJ (1995) Reduced expression of retinoblastoma (Rb) gene protein in related to cell proliferation and prognosis in transitional cell bladder cancer. *J Cancer Res Clin Oncol* **121**: 44–50

Livingstone L, White A, Sprouse J, Livanos E, Jacks T and Tlste TD (1992) Altered cell cycle arrest and gene amplification potential accompanied loss of wild-type *TP53*. *Cell* **70**: 923–935

Lowe SW, Schmitt EM, Smith SW, Osborne BA and Jacks T (1993) p53 is required for radiation-induced apoptosis in mouse thymocytes. *Nature* **362**: 847–849

Lunec J and Mellon JK (1994) Molecular biology and bladder cancer. In *Tumours in urology*, Neal DE (ed.) pp. 19–45. Springer-Verlag: London

Mazars GR, Jeanteur P, Lynch HT, Lenoir G and Theillet C (1992) Nucleotide sequence polymorphism in a hotspot mutation region of the *TP53* gene. *Oncogene* **7**: 781–782

Mostofi FK (1973) *International Histological Classification of Tumours. Histological Typing of Urinary Bladder Tumours*, volume 10. WHO: Geneva

Neal DE, Sharples L, Smith K, Fennelly J, Hall RR and Harris AL (1990) The epidermal growth factor receptor and the prognosis of bladder cancer. *Cancer* **65**: 1619–1625

Office of Population Census Statistics (1993) *Cancer Statistics*. London: HMSO

Pavletich NP, Chambers KA and Pabo CO (1993) The DNA-binding domain of TP53 contains the four conserved regions and the major mutation hotspots. *Genes Dev* **7**: 2556–2564

Peto R, Pike MC, Armitage P, Breslow NE, Cox DR, Howard SV, Mantel N, McPherson K, Peto J and Smith PG (1977) Design and analysis of randomised clinical trials requiring prolonged observation of each patient. *Br J Cancer* **35**: 1–39

Rodrigues NR, Rowan A, Smith MEF, Kerr IB, Bodmer WF, Cannon JV and Lane DP (1990) *TP53* mutations in colorectal cancer. *Proc Natl Acad Sci* USA **87**: 7555–7559

Sidransky D, Eschenbach AV, Tsai YC, Jones P, Summerhayes I, Marshall F, Paul M, Green P, Hamilton SR, Frost P and Vogelstein B (1991) Identification of *TP53* gene mutations in bladder cancers and urine samples. *Science* **252**: 706–709

Sjogren S, Inganas M, Norberg T, Lindgren A, Nordgren H, Holmberg L and Bergh J (1996) The *TP53* gene in breast cancer: prognostic value of complementary DNA sequencing versus immunohistochemistry. *J Natl Cancer Inst* **88**: 3/4, 173–182

Spruk III CH, Rideout III WM, Olumi AF, Ohneseit PF, Yang AS, Tsai YC, Nichols PW, Horn T, Hermann GG, Steven K, Ross RK, Yu MC and Jones PA (1993) Distinct pattern of *TP53* mutations in bladder cancer: relationship to tobacco usage. *Cancer Res* **53**: 1162–1166

Takahashi T, Suzuki H, Hida T, Sekido Y, Ariyoshi Y and Ueda R (1991) The *TP53* gene is very frequently mutated in small-cell lung cancer with a distinct nucleotide substitution pattern. *Oncogene* **6**: 1775–1778

Uchida T, Wada C, Ishida H, Wang C, Egawa S, Yokoyama E, Kameya T and Koshiba K (1995) *TP53* mutations and prognosis in bladder tumours. *J Urol* **153**: 1097–1104

Union Internationale Contre le Cancer (1978) TNM *Classification of Malignant Tumours, Bladder*, 3rd edn, pp. 113–117

Vageli D, Kiaris H, Delakas D, Anezinis P, Cranidis A and Spandidos DA (1996) Transcriptional activation of H-ras, K-ras and N-ras proto-oncogenes in human bladder tumours. *Cancer Lett* **107**: 241–247

Vet JAM, Bringuier PP, Poddighe PJ, Karthaus HFM, Debruyne FMJ and Schalken JA (1994) *TP53* mutations have no additional prognostic value over stage in bladder cancer. *Br J Cancer* **70**: 496–500

Vet JA, Bringuier PP, Schaafsma HE, Witjes JA, Debruyne FM and Schalken JA (1995) Comparison of TP53 protein overexpression with *TP53* mutation in bladder cancer: clinical and biological aspects. *Lab Invest* **73**: 837–843

Zhan O, Carrier F and Fomace AJ (1993) The *gadd* and *myD* genes define a novel set of mammalian genes encoding acidic proteins that suppress cell growth. *Mol Cell Biol* **13**: 4242–4250

*© Cancer Research Campaign 1998*